# ^18^F-DOPA uptake illustration acts as an indicator for renal sympathetic activity

**DOI:** 10.3389/fphys.2025.1569699

**Published:** 2025-10-20

**Authors:** Rui Wu, Saijian Shi, Yuanjie Huang, Feng Tian, Jiqin Hou, Xiangqing Kong, Longxiang Sheng, Tao Zhang, Lijun Tang, Wei Sun

**Affiliations:** ^1^ Department of Cardiology, The First Affiliated Hospital with Nanjing Medical University, Nanjing, China; ^2^ Department of Nuclear Medicine, The First Affiliated Hospital with Nanjing Medical University, Nanjing, China; ^3^ Department of Radiopharmaceuticals, School of Pharmacy, National Center of Technology Innovation for Biopharmaceuticals, Innovation Center of Suzhou Nanjing Medical University, Nuclear Medicine Clinical Translation Center, Northern Jiangsu Institute of Clinical Medicine, Nanjing Medical University, Nanjing, China; ^4^ Department of Cardiology, The Affiliated Suzhou Hospital of Nanjing Medical University, Suzhou Municipal Hospital, Gusu School, Nanjing Medical University, Suzhou, Jiangsu, China

**Keywords:** hypertension, sympathetic nerve, renal denervation, positron emission tomography-computed tomography, L-Dopa

## Abstract

Renal denervation (RDN) has emerged as a promising therapeutic approach for hypertension, particularly in patients with refractory hypertension. However, non-invasive measurement of the degree of renal nerve ablation has still been a challenge. This study aims to develop a novel method for assessing the efficacy of RDN. Based on ^18^F-DOPA micro PET-CT imaging, we demonstrate that bilateral RDN significantly reduces ^18^F-DOPA uptake in the kidneys of spontaneously hypertensive rats (SHRs) with normal renal function. A similar trend was observed in Wistar Kyoto rats (WKYs). Meanwhile, the effectiveness of RDN was further validated through blood pressure (BP) monitoring, Western blot analysis of sympathetic nerve markers, and immunohistochemistry of nerves. In conclusion, our data suggest that ^18^F-DOPA PET-CT imaging is parallel with the sympathetic nerve activity of kidney which provides valuable insights for *in vivo* evaluating the efficacy of RDN and may serve as a critical tool for clinically assessing its therapeutic outcomes. The underlying molecular mechanisms responsible for the observed reduction in renal ^18^F-DOPA uptake in SHRs following RDN warrant further investigation.

## Introduction

Hypertension is the most common cardiovascular disorder and is a leading cause of damage to multiple target organs. Primary hypertension is strongly associated with increased sympathetic nervous system activity. The renal sympathetic nervous system plays a crucial role in regulating both blood pressure (BP) and renal function (Osborn et al., 2021). Renal denervation (RDN) is a novel therapeutic approach for hypertension that involves the use of radiofrequency ablation or ultrasound catheters to ablate the renal sympathetic nerves. By reducing sympathetic nervous system activity, RDN decreases renal vascular resistance, renin release, and sodium reabsorption, thereby lowering BP (Mahfoud et al., 2021; Kusayama et al., 2024). With ongoing optimization of radiofrequency ablation systems and the validation of numerous clinical studies, RDN has emerged as a safe and effective treatment for hypertension (Weber et al., 2019; Rey-García and Townsend, 2022). Although several studies suggest that RDN may reduce the occurrence of cardiovascular events, research on the long-term prognosis of RDN in cardiovascular events is still in its early stages. Further studies are needed to clarify this point (Wang et al., 2024; Mahfoud et al., 2022). However, aside from indirect methods, such as monitoring postoperative blood pressure variations, there is currently no direct approach to assess the extent of ablation during or after the procedure (Nair et al., 2016). Therefore, it is crucial to develop methods for improving the success rate of ablation and identifying indicators to evaluate the immediate effects of RDN.

Levodopa (L-DOPA) serves as a precursor to catecholamine neurotransmitters. Within the central nervous system (CNS), tyrosine is initially converted to L-DOPA through the action of tyrosine hydroxylase (TH), which is subsequently decarboxylated by aromatic L-amino acid decarboxylase (AADC) to form dopamine. Dopamine is either stored in synaptic vesicles or released from the presynaptic membrane. Upon release, dopamine interacts with dopamine receptors (DR) located on both the presynaptic and postsynaptic membranes, mediating a variety of physiological responses. The kidneys, in addition to the CNS, are a significant site of dopamine production. Renal dopamine synthesis primarily occurs through the uptake of circulating L-DOPA, facilitated by both sodium-dependent and sodium-independent transport systems (Pinto et al., 2013). Within the sodium-independent pathway, the type 2 L-type amino acid transporter (LAT2) plays a critical role in the uptake of L-DOPA by renal epithelial cells. Overexpression of LAT2 in the kidneys of spontaneously hypertensive rats (SHR) has been shown to enhance the absorption of L-DOPA, suggesting a potential mechanism for increased dopamine synthesis in this model (Pinho et al., 2003). Labeling L-DOPA with the radioactive isotope ^18^F on the sixth carbon atom forms radiolabeled L-DOPA analogs, which can be visualized using positron emission tomography (PET). Initially, ^18^F-DOPA PET imaging was employed for diagnosing Parkinson’s disease, where it allows for the detection of dopaminergic neuron degeneration in the substantia nigra and striatum (Sawle, , 1993). Recently, ^18^F-DOPA PET has emerged as a key diagnostic tool for imaging neuroendocrine tumors, with broad clinical applications in conditions like carcinoid tumors, insulinomas, pheochromocytomas, paragangliomas, and medullary thyroid cancer (Jager et al., 2008).

Given the significant uptake and metabolism of L-DOPA by renal tissues, coupled with evidence suggesting a reduction in catecholamine levels within the kidneys following RDN (Pires et al., 2015), we hypothesize that ^18^F-DOPA PET can serve as an effective imaging probe for assessing renal sympathetic nervous activity. The team’s previous research found that RDN can reduce renal sympathetic nerve activity and norepinephrine (NE) content in the kidneys of hypertensive rats (Li et al., 1985). In this study, we observed that ^18^F-DOPA exhibited strong retention in the kidneys after metabolic stabilization. In SHRs, renal uptake of ^18^F-DOPA was significantly reduced following RDN, which may reflect changes in renal sympathetic nervous system activity. Therefore, ^18^F-DOPA PET-CT could serve as an effective tool for assessing the efficacy of RDN in the future.

## Methods

### Animal model

All rats, obtained from Beijing Weitong Lihua Experimental Animal Technology Co., China, were kept in a standard SPF environment (room temperature: 24 °C; relative humidity: 55%; 12 h of alternating brightness and darkness). Animals have free access to food and water. All animal experiments were conducted in accordance with the relevant regulations of the Experimental Animal Welfare Ethics Committee of Nanjing Medical University. experiments were conducted according to the National Research Council’s guidelines.

^18^F-DOPA micro positron emission tomography-computed tomography (^18^F-DOPA micro PET-CT) scanning.

The ^18^F-fluoride ion was generated using a GE PETtrace800 cyclotron. Subsequently, it was employed in the AllinOne synthesis module with a commercial chemical reagent and an ^18^F-DOPA cassette and kit (Paite Biotechnology Company, China), to ultimately produce ^18^F-DOPA. The radiochemical purity of ^18^F-DOPA exceeded 95% as determined by HPLC, and the radiochemical synthesis yield was 40% ± 5% after decay correction.

MicroPET imaging was performed using a micro-PET/CT system (SuperNova, PINGSENG Healthcare, China). Rats were anesthetized with isoflurane (1.5%–2.5% maintained concentration) (RWD Life Science, China), and 1 mCi of ^18^F-DOPA, dissolved in saline, was injected into their tail veins. PET-CT scanning was initiated 60 min post-injection to observe the uptake of ^18^F-DOPA in the kidneys across different groups. To further investigate the dynamic distribution of ^18^F-DOPA *in vivo*, PET-CT scans were also conducted at 15, 60, and 180 min after injection. Throughout the procedure, the rats inhaled isoflurane continuously to maintain the anesthetic effect. Each scanning bed position was acquired for 10–30 min. The scanning parameters were set as follows: an energy window of 350–650 keV, tube voltage of 30–90 kVp, and exposure time of ≤10 s. The total and residual doses were meticulously measured before and after injection. Each rat was individually numbered and weighed to ensure accurate quantification of organ standard uptake values (SUV).

### Renal denervation and sham surgery

After 1 week of adaptive feeding in rats, rats were devided into 4 groups: WKY-Sham; WKY-RDN; SHR-Sham; SHR-RDN. All rats were fasted for 12 h prior to surgery but were allowed free access to water. Prior to the surgery, the rats were placed in an induction chamber for anesthesia. Once the rats were fully anesthetized, they were removed from the chamber, and a mask was placed over their nose and mouth for continuous inhalation of 2.5% isoflurane. The abdominal area of the rats was shaved, and the skin was disinfected. For the RDN groups, a midline laparotomy was performed to expose the renal vessels and renal sinus. Using forceps, the renal arteries and veins were separated, and visible nerves were dissected. Renal arteries and hilum were applied 10% phenol/ethanol solution repeatedly for bilateral renal artery ablation. After completion, the abdominal cavity was rinsed with physiological saline and the abdomen was closed with 3-0 suture. The sham-operated rats also underwent laparotomy under anesthesia to expose the kidneys and renal vasculature, but the 10% phenol/ethanol solution was not applied for renal denervation.

### High salt diet induced hypertension in sprague-dawley (SD) rats

After entering the barrier environment, all rats were adaptively fed with regular feed for 1 week. After 1 week of adaptive feeding, the SD rats were randomly divided into two groups: Control Diet (CD) and High-Salt Diet (HSD) groups. The rats in the HSD group were switched from a regular diet to a high-salt diet (Jiangsu Xietong Pharmaceutical Bio-engineering Co., China) and were continued on the diet for 6 weeks.

## BP measurement

Under physiological conditions, WKY and SHR rats underwent their first ^18^F-DOPA Micro PET-CT scan, followed by tail arterial pressure measurement. Blood pressure changes were monitored continuously for 2 weeks after either the sham surgery or RDN procedure. For the CD and HSD rats, blood pressure measurements were taken at the end of the modeling period.

Before blood pressure measurement, the temperature of the animal platform was preheated to 36 °C. The rats were then placed in the restrainer on the animal platform and allowed to rest for 8–10 min to stabilize their condition. Once stable, a rubber cuff was placed around the base of the rat’s tail and fixed to the animal platform. The temperature of the platform was maintained at 36 °C during the measurement. After the procedure, the animals were removed from the restrainer and returned to their cages.

### Tissue dissections

Rats peripheral blood samples were collected from the orbital venous plexus under deep anesthesia with isoflurane. All rats transcardially perfused with phosphate buffered saline (PBS) until the liver turned from red to white. Subsequently, the renal artery and kidney were cut off. The collected tissue was quickly fixed in 4% paraformaldehyde (PFA) or frozen in liquid nitrogen and stored at −80 °C for further analysis.

### Serum renal function test

After collection, the rat blood samples were left to stand at room temperature for 2 h. They were then placed in a refrigerated high-speed centrifuge at 4 °C and centrifuged at 3,000 rpm for 15 min. After centrifugation, the supernatant serum was transferred to a new EP tube and stored at −80 °C. The serum renal function test was conducted by Wuhan Sevier Biotechnology Company according to commercial procedures.

### Protein extraction and western blot analysis

A portion of kidney tissue was homogenated in RIPA lysis buffer (Beyotime Biotechnology Co., China). The supernatant was obtained after centrifugal at 12000 rpm for 15 min at 4 °C. SDS-PAGE protein loading buffer was added and the protein was boiled for 5 min at 95 °C–100 °C. 10% SDS-PAGE fast gel (Yase Biomedical Technology Co.,China) (30ug tissue/swimming lane) was used for electrophoresis and transferred to polyvinylidene fluoride (PVDF) membrane. 5% bovine serum albumin (BSA) was used for blocking for 2 h at room temperature. The membranes were incubated overnight for 12–16 h with TH (1:5,000, proteintech); GAPDH (1:10,000, proteintech). The next day, the membranes were incubated with the corresponding secondary antibody at room temperature for 2 h. The protein was visualized by enhanced chemiluminescence method. Using ImageJ to quantify the grayscale values of the stripes.

### Immumohistochemical staining

Approximately 5 μm paraffin embedded tissue sections were used for immunohistochemical staining. The sections were incubated overnight at 4 °C with primary antibody TH (1:6,000, proteintech) for 12–16 h. Then, a secondary antibody (1:20,000, abcam) bound to horseradish peroxidase (HRP) was used. The sections were Incubated in DAB solution (Sevier Biotechnology Co., China) for 3 min, counterstained with hematoxylin for 5 min. Images were obtained using a microscope (Zeiss, Germany).

### Statistic analysis

The SUV of the kidneys obtained from ^18^F-DOPA micro PET-CT scanning was analyzed by Professor Zhang Tao’s team from Nanjing Medical University using a double-blind method. The team was not aware of all surgical procedures and purposes in rats beforehand. We employed an image anonymization method (using image numbers instead of original labels) to ensure the independence of the analysts. This approach helps eliminate any potential bias during the analysis process and strengthens the integrity of the results.

Amide software was used to accurately locate the left and right kidneys and their cortex and medulla regions through CT imaging, including transverse, coronal, and sagittal planes. The left and right kidneys and their medulla were delineated using Ellipsoid ROI, as they exhibit regular ellipsoidal shapes. The renal cortex was outlined using 3D freehand ROI. The dimensions of ROI parameters include voxel size in x, y, and z directions, with calculations performed over all voxels and ROI mean values. Although minor variations in ROI delineation may occur between different operators, they do not affect the comparisons between experimental groups or the overall trends of the final results.

All quantitative values of the results are expressed as mean ± standard deviation (SD), representing data from at least three independent experiments. Data between two groups were analyzed by Student’s t-test. Significant differences between three or more groups were determined through ANOVA multiple comparison test. A P-value less than 0.05 is considered statistically significant. All statistical calculations were performed using GraphPad Prism software.

## Results

### ^18^F-DOPA has good retention characteristics in kidneys

**FIGURE 1:**
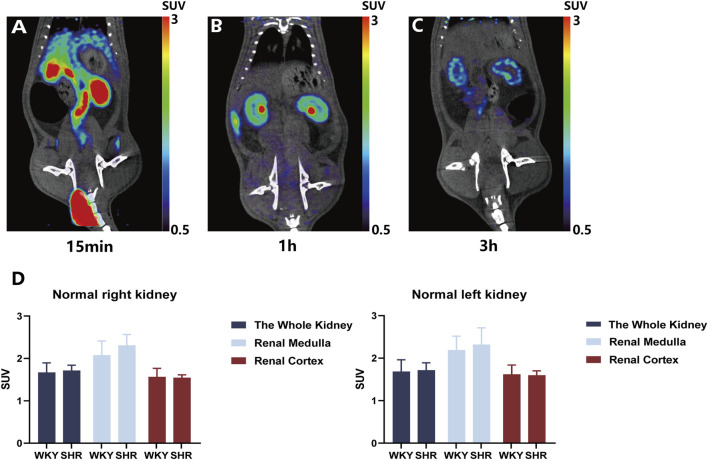
Renal uptake of ^18^F-DOPA visualized through ^18^F-DOPA microPET-CT imaging. **(A–C)** Representative ^18^F-DOPA microPET-CT images in rats. Images were acquired at three time points following the injection of ^18^F-DOPA: 15 min **(A)**, 1 h **(B)**, and 3 h **(C)**. The kidneys of rats show prominent uptake of ^18^F-DOPA at all time points. **(D)** Statistical analysis of renal ^18^F-DOPA uptake in WKYs and SHRs 1 h post-injection. No significant difference was observed in the SUV of ^18^F-DOPA across different regions of the kidneys in either group. WKY: n = 8, SHR: n = 8. Statistical analysis was performed using a Student’s t-test. Data are presented as mean ± SD.

^18^F-DOPA exhibits favorable retention characteristics in the kidneys. To determine the physiological distribution and the optimal time for analyzing ^18^F-DOPA uptake in rat kidneys, a 1 mCi dose of ^18^F-DOPA was administered via tail vein injection to all rats. Micro PET-CT scans were performed at 15 min (Figure 1A), 1 h (Figure 1B), and 3 h (Figure 1C) post-injection. At 1 h post-injection, ^18^F-DOPA uptake was minimal in organs other than the kidneys, with no significant retention observed in the ureters. Additionally, PET-CT imaging clearly distinguished the medulla and cortex of the rat kidneys at this time point (Figure 1B). ^18^F-DOPA remained in the kidneys even after 3 h post-injection (Figure 1C), demonstrating its strong retention characteristics in this organ. Based on this observed retention, we sought to explore whether ^18^F-DOPA could reflect renal sympathetic nervous activity. In physiological conditions, the renal efferent nerve activity of WKY rats is similar to that of SHR rats (Franc et al., 1981), we performed baseline micro PET-CT scans on WKYs and SHRs. Data analysis revealed no significant differences in ^18^F-DOPA uptake between WKYs and SHRs in the right kidney, right renal cortex, right renal medulla, left kidney, left renal medulla, and left renal cortex under normal conditions (Figure 1D).

### RDN effectively reduces sympathetic nerve activity

**FIGURE 2:**
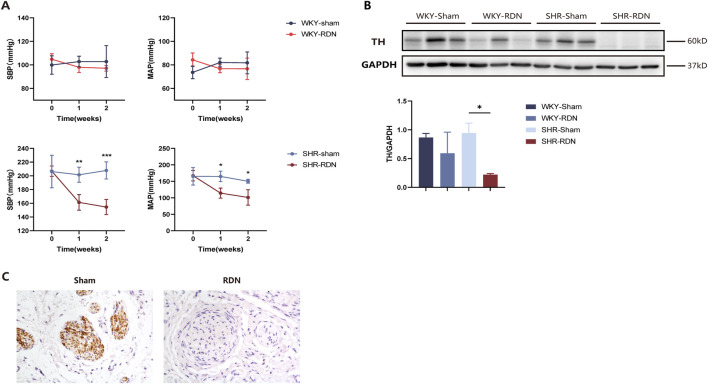
RDN effectively reduces sympathetic nerve activity. **(A)** Impact of bilateral RDN on blood pressure in WKY and SHR rats. Compared to SHR-Sham, bilateral RDN resulted in a sustained reduction in SBP and MAP in SHR-RDN rats within 2 weeks after the procedure. No significant change in BP was observed between WKY-RDN and WKY-Sham rats. Each group: n = 4. *P < 0.05, **P < 0.01, ***P < 0.001, ANOVA. Data are presented as mean ± SD. **(B)** Expression of TH protein in rat kidney tissue following RDN or Sham surgery. Western blot analysis of kidney tissue showed that, 2 weeks after bilateral RDN, the expression of TH in the kidneys of SHR-RDN rats was significantly reduced compared to SHR-Sham, while no significant change in TH expression was observed in WKY-RDN rats compared to WKY-Sham. Similarly, there was no difference in the expression of TH protein in the kidneys between the WKY-Sham and SHR-Sham. Each group: n = 3, *P < 0.05, ANOVA. Data are presented as mean ± SD. **(C)** TH immunohistochemical staining of renal artery tissue. Immunohistochemistry revealed successful ablation of sympathetic nerve fibers around the renal artery in SHR-RDN rats following the RDN procedure, as indicated by a reduction in TH-positive staining. Each group: n = 3.

After performing baseline ^18^F-DOPA micro PET-CT scans, rats were divided into four groups: WKY-Sham, WKY-RDN, SHR-Sham, and SHR-RDN. Following baseline BP measurement, all rats underwent either RDN or Sham surgery, and BP was continuously monitored for 2 weeks post-surgery. BP measurements revealed that, within 2 weeks after RDN, both systolic blood pressure (SBP) and mean arterial pressure (MAP) in the SHR-RDN group continuously decreased. However, no significant difference in BP was found between the WKY-Sham and WKY-RDN groups (Figure 2A).

Western blot analysis of renal tissue and immunohistochemical staining of renal artery showed that RDN effectively reduced renal sympathetic nerve activity. Compared with SHR-Sham, the expression of TH protein in the renal tissue of SHR-RDN was significantly reduced (Figure 2B), The representative image of TH immunohistochemistry staining is shown in Figure 2C. According to the semi-quantitative scoring criteria (Sakakura et al., 2014), we found that samples with weak or no TH staining were predominant in the SHR-RND group, suggesting that our method effectively inhibits renal neuroactivity, demonstrating a therapeutic effect. However, no significant difference in TH expression was observed between the WKY-RDN and WKY-Sham groups (Figure 2B), Similarly, there was no difference in the expression of TH protein in the kidneys between the WKY-Sham and SHR-Sham groups (Figure 2B). These data demonstrate that under normal physiological conditions, the renal sympathetic activity in WKY rats is similar to that in SHR rats. Therefore, when subjected to ^18^F-DOPA PET-CT scanning in the baseline state, there is no significant difference in renal uptake of ^18^F-DOPA between the two rat strains. However, after RDN surgery, the renal sympathetic activity in SHR rats was significantly reduced.

### Renal uptake of ^18^F-DOPA decreased in SHR-RDN

**FIGURE 3:**
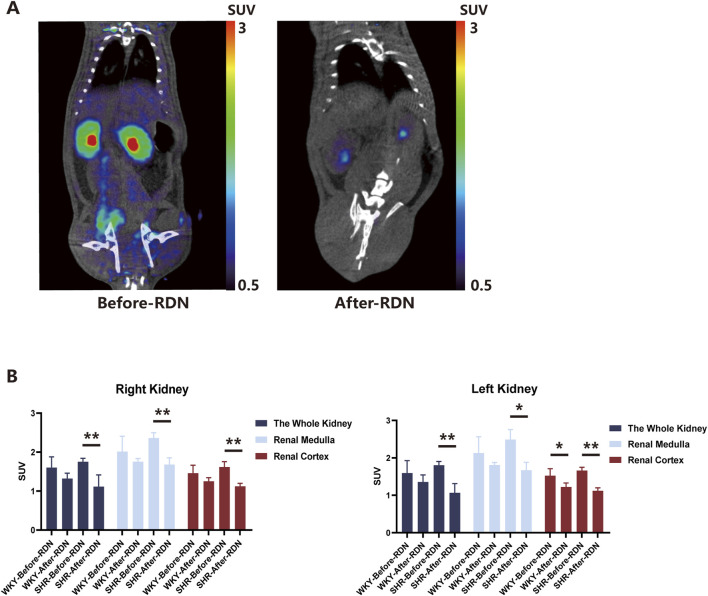
Renal uptake of ^18^F-DOPA in WKYs and SHRs before and after RDN. **(A)** Representative ^18^F-DOPA microPET-CT images. Images demonstrate the renal uptake of ^18^F-DOPA in SHR rats before and 2 weeks after bilateral RDN. Notable differences in the uptake of ^18^F-DOPA within the kidneys are observed pre- and post-RDN. **(B)** Effect of RDN on ^18^F-DOPA uptake in SHR and WKY rats. Following RDN, a significant reduction in ^18^F-DOPA uptake was observed across various regions of the kidneys in SHR-RDN rats. A decreasing trend in ^18^F-DOPA uptake was also noted in WKY-RDN rats, though the change was less pronounced. It is important to note that cortical SUV data for one SHR-RDN rat was excluded due to a lack of visible cortical uptake. Each group: n = 4. *P < 0.05, **P < 0.01, one-way ANOVA. Data are presented as mean ± SD.

Two weeks after either sham surgery or bilateral RDN, ^18^F-DOPA micro PET-CT scans were performed on all four groups of rats under the same conditions, and the SUV obtained were compared with pre-RDN levels for each corresponding group. We found that bilateral RDN significantly reduced ^18^F-DOPA uptake in the kidneys of SHR-RDN rats. Specifically, the right kidney, right renal medulla, right renal cortex, left kidney, left renal medulla, and left renal cortex of SHR-RDN all showed significant decreases in ^18^F-DOPA SUV. In contrast, while there was a trend toward reduced SUV in various regions of the kidneys in WKY-RDN rats, these changes were not statistically significant (Figure 3B). Representative ^18^F-DOPA micro PET-CT images of renal uptake before and 2 weeks after RDN in SHRs are shown in Figure 3A. The renal SUV in the WKY-sham and SHR-sham showed no significant change compared to pre-surgical levels (Supplementary Figure S1B), further excluding the potential impact of fat removal from the renal sinus during the RDN surgery on the renal uptake of ^18^F-DOPA in rats. Supplementary Figure S1A presents representative MicroPET-CT images of renal ^18^F-DOPA uptake in SHR rats before and after sham surgery.

Due to surgical factors, some rats exhibited abnormal renal imaging on CT (Supplementary Figure S1C), which were also consistent with observed renal enlargement upon dissection. and thus only rats with normal bilateral kidney imaging were selected for effective data analysis. In the micro PET-CT scans, rats with normal bilateral kidney images showed no abnormalities in serum renal function tests. The levels of creatinine (CREA) and urea nitrogen (BUN) in the blood of RDN rats were not significantly different from those of Sham group rats (Supplementary Figure S2A).

### Elevated sympathetic activity was observed in the kidneys of rats fed a high-salt diet

RDN is a surgical technique that reduces sympathetic nerve activity in the kidneys and lowers blood pressure. Previous studies have demonstrated that a long-term high-salt diet enhances sympathetic nerve activity in both the paraventricular nucleus and the kidneys of rats (Yu et al., 2019; Su et al., 2021). To investigate whether ^18^F-DOPA can effectively reflect renal sympathetic activity, we selected a model known to enhance renal sympathetic activity—the high-salt diet-induced hypertension model.

**FIGURE 4:**
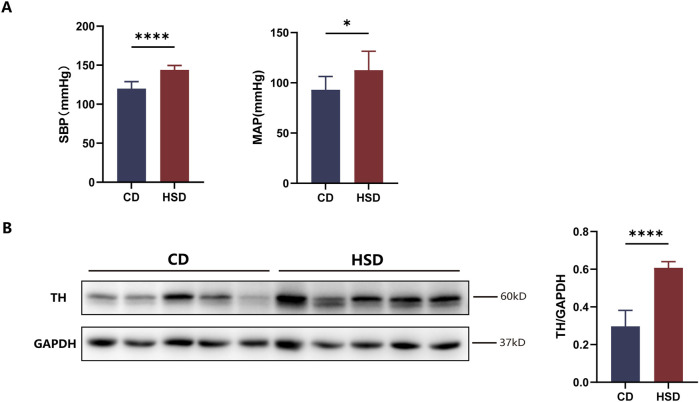
Activation of renal sympathetic nervous system in HSD rats. **(A)** Effect of high-salt feeding on blood pressure. After 6 weeks of high-salt feeding, both SBP and MAP were significantly elevated in HSD rats compared to CD rats. Each group: n = 8, *P < 0.05, ****P < 0.0001, Student’s t-test. Data are presented as mean ± SD. **(B)** Expression of TH in renal tissue. After 6 weeks of high-salt feeding, the expression of TH, a marker of sympathetic nerve activity, was significantly higher in HSD rats compared to CD. Each group: n = 5. ****P < 0.0001, Student’s t-test. Data are presented as mean ± SD.

After feeding SD rats a high-salt diet for 6 weeks, we observed a increase in SBP and MAP in the high-salt diet (HSD) group compared to the control diet (CD) group (Figure 4A). Additionally, Western blot analysis revealed a significant increase in TH protein expression in the renal tissue of HSD rats (Figure 4B). These findings suggest that, after 6 weeks of high-salt feeding, renal TH expression was upregulated, indicating activation of the renal sympathetic nervous system. However, while serum BUN levels were elevated in the HSD group, CREA and urea (UREA) levels did not show significant changes (Supplementary Figure S2B).

### Renal uptake of ^18^F-DOPA increased in HSD rats

**FIGURE 5:**
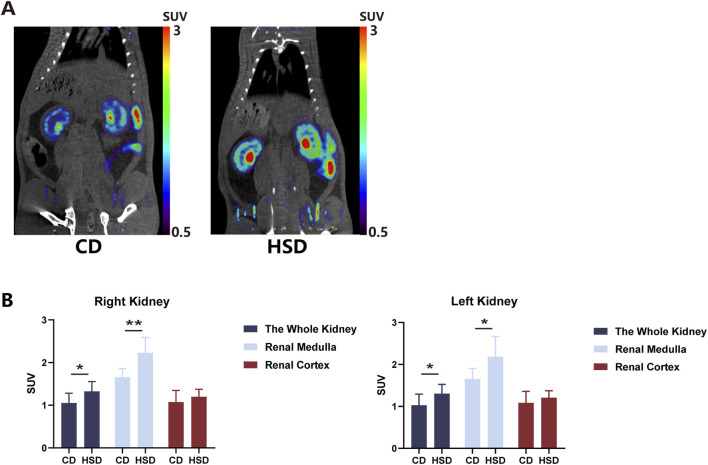
High salt diet increased ^18^F-DOPA uptake in rat kidneys. **(A)** Representative ^18^F-DOPA microPET-CT images of kidneys from CD and HSD rats. Images show the renal uptake of ^18^F-DOPA in kidneys from CD and HSD rats. The visual differences in uptake highlight the effects of the high-salt diet on renal sympathetic activity. **(B)** Statistical analysis of the SUV of ^18^F-DOPA in the kidneys of CD and HSD rats. After 6 weeks of high-salt feeding, the kidneys of HSD rats showed significantly increased uptake of ^18^F-DOPA compared to CD rats. This change paralleled the alteration in renal sympathetic activity. Each group: n = 8. *P < 0.05, **P < 0.01, Student’s t-test. Data are presented as mean ± SD.

After feeding SD rats a high-salt diet for 6 weeks, rats from both the CD and HSD groups were injected with 1 mCi of ^18^F-DOPA via the tail vein under identical conditions, and micro PET-CT scans were performed 1 h later. The results demonstrated that the high-salt diet increased renal uptake of ^18^F-DOPA in SD rats (Figure 5B), Compared to CD rats, the uptake of ^18^F-DOPA in the left and right kidneys as well as the renal medulla of HSD rats was significantly increased, while no statistical significance was observed in the cortical region (Figure 5B). This finding suggests that a long-term high-salt diet may increase renal sympathetic nerve activity, thereby enhancing the uptake of ^18^F-DOPA in the kidneys as a reflection of sympathetic nervous activity. Figure 5A shows representative micro PET-CT images of ^18^F-DOPA uptake in the kidneys of CD and HSD rats.

### RDN and high-salt diet do not alter the dopamine reuptake capacity of renal dopaminergic neurons

DAT is a key membrane protein widely found in various regions of the brain, especially those associated with emotional regulation, the reward system, motor control, and neurotransmission. It regulates the levels of dopamine in the synaptic cleft through a reuptake mechanism, which is crucial for maintaining the brain’s neurochemical balance (Chen and Reith, 2000). Neither the 2-week RDN nor the 6-week high-salt diet resulted in a significant change in the expression of DAT in renal tissue (Supplementary Figure S3). This suggests that the RDN surgery or high-salt diet may not regulate synaptic dopamine levels by altering the reuptake capacity of dopaminergic neurons, and thus may not affect sympathetic nervous activity in this way.

## Discussion

In this study, we found that following successful RDN, the renal ^18^F-DOPA SUV in SHR-RDN rats significantly decreased 1 h after injection of ^18^F-DOPA, compared to pre-surgery levels. A similar trend was also observed in the WKY-RDN group. When rats were fed a high-salt diet to activate renal sympathetic nervous system activity, renal uptake of ^18^F-DOPA increased. These findings suggest that ^18^F-DOPA can, to some extent, reflect the activity of the renal sympathetic nervous system. Therefore, ^18^F-DOPA PET-CT may have potential clinical value in assessing the success of RDN procedures. In the Micro PET-CT scans, some rats exhibited significant unilateral or bilateral renal enlargement due to surgical reasons, which was clearly visible during imaging. Post-mortem dissection also confirmed the presence of abnormal renal enlargement in these rats. Therefore, we excluded the data obtained from scanning these rats from the statistical analysis, as it was considered unreliable due to the observed anatomical abnormalities. Except for the rats with significant renal imaging abnormalities mentioned above, all other experimental rats were included in the statistical analysis. It is noteworthy that under our stringent experimental conditions, the uptake of ^18^F-DOPA in the kidneys of each SHR-RDN rat was significantly reduced. Additionally, a sham surgery group was included as a negative control to ensure the reliability of the results. This approach ensures that only data from rats without such abnormalities were included in the analysis, maintaining the validity of the study’s conclusions.

The key potential translational value of ^18^F-DOPA dependent PET-CT imaging might be used in evaluating the ablation efficacy of renal denervation therapy by RFCA catheters. Even several RDN device are approved by FDA for clinical practice, there is absent of non-invasive assessment for the efficacy of nerve ablation. Our technology allows for non-invasive and quantitative assessment of renal sympathetic nerve activity, providing direct and objective evaluation metrics for RDN outcomes. Secondly, ^18^F-DOPA PET-CT has high spatial resolution and sensitivity. Furthermore, this technique is repeatable, facilitating long-term follow-up and dynamic monitoring. Compared to microscopic nerve angiography, this method is safer. Since the development of microscopic nerve angiography, it has been shown to be associated with various acute or chronic adverse events (Meah et al., 2019). In contrast, the radioactive agent ^18^F-DOPA has been used for the diagnosis of population-based diseases for many years, and its safety has been widely demonstrated. Baroreceptor sensitivity is a method used to determine the function of the baroreceptor reflex. There is a reference threshold for baroreceptor sensitivity in different populations (Tank et al., 2000), but individual differences between populations are often significant. By performing ^18^F-DOPA scans before and after RDN, and comparing the individual data pre- and post-surgery, we can address this issue.

However, ^18^F-DOPA PET-CT also has some limitations. Firstly, the high cost of the procedure may limit its widespread clinical application. Secondly, the short half-life of ^18^F (approximately 110 min) necessitates the proximity of a cyclotron, which may be difficult to implement in certain regions. Additionally, image interpretation requires specialized training, which may introduce a degree of subjectivity. The primary focus of this study is the manual segmentation of murine organs for the analysis of SUV. In recent years, artificial intelligence (AI) technology has made significant advancements in the field of medical imaging, particularly in automating image segmentation and enhancing image processing accuracy (Iqbal et al., 2021; Iqbal et al., 2020; Iqbal et al., 2022). The incorporation of AI-driven methodologies could potentially streamline the workflow in future research, significantly improving the efficiency of organ delineation and quantitative analysis. This advancement has the potential to reduce inter-observer variability, accelerate data processing, and enhance the overall precision of SUV measurements in preclinical imaging studies.

In this study, we acknowledge that the histological evaluation following renal artery ablation was not comprehensive. Specifically, we did not assess the extent of nerve bundle damage or the density of nerve fibers around the renal artery. In addition, a limitation of this study is that, due to the cost of obtaining the ^18^F-DOPA radiopharmaceutical and the expense of small animal Micro PET-CT scans, we were unable to validate the sensitivity of ^18^F-DOPA PET-CT imaging for reflecting renal sympathetic nerve activity in more hypertensive animal models receiving RDN treatment. Therefore, future studies are recommended to further explore and validate these results in different animal models or human cohorts.

An growing body of evidence suggests that RDN can not only treat hypertension but also improve outcomes in patients with severe chronic kidney disease (CKD) (Lo et al., 2024), enhance left ventricular (LV) function in heart failure (HF) patients (Fukuta et al., 2017), and reduce the incidence of ventricular fibrillation associated with heart failure (Yamada et al., 2020). Studies have shown that RDN can lead to significant BP reduction in most patients, with this reduction being safely maintained for at least 10 years after the procedure (Wang et al., 2024; Panchavinnin et al., 2022). Given its potential to become a key therapeutic approach for cardiovascular diseases, there is an pressing need for a reliable method to assess the extent of renal nerve ablation during or after the procedure. ^18^F-DOPA PET-CT scanning could be an effective tool for clinicians to evaluate the success of RDN treatment.

However, the exact mechanism underlying the effectiveness of RDN in treating hypertension remains unclear. Beyond its role in reducing the impact of the sympathetic nervous system on renal vascular resistance, renin release, and sodium reabsorption, recent studies have also shown that RDN can prevent T cell infiltration and subsequent kidney damage (Xiao et al., 2015).

In this study, the mechanisms underlying the decreased renal uptake of ^18^F-DOPA observed in MicroPET-CT imaging following RDN, as well as the increased renal uptake of ^18^F-DOPA after a high-salt diet, have not been fully elucidated. However, it is noteworthy that the reduction in renal sympathetic nerve activity caused by RDN and the increase in renal sympathetic nerve activity induced by a high-salt diet are not due to changes in the renal dopaminergic neurons' ability to reuptake dopamine.

However, we believe that the high activity of AADC in peripheral tissues cannot fully explain the retention of ^18^F-DOPA in the kidneys seen on micro PET-CT scans. Although ^18^F-DOPA is excreted via the urinary tract, its hydrophilic properties lead to rapid metabolism and clearance to the bladder. Moreover, in both normal humans and animals, brain uptake of ^18^F-DOPA is typically not observed on PET-CT scans unless peripheral AADC activity is inhibited by pre-treatment with carbidopa. Without carbidopa, peripheral AADC rapidly metabolizes ^18^F-DOPA, preventing its entry into the brain. Only when carbidopa is administered does the peripheral metabolism of ^18^F-DOPA decrease, allowing it to cross the blood-brain barrier (Chondrogiannis et al., 2013). Based on these principles, we speculate that the decrease in ^18^F-DOPA SUV observed on micro PET-CT in rats after RDN may result from the following: AADC is highly expressed in the proximal tubules of the kidney. As a key enzyme catalyzing the conversion of L-DOPA to dopamine, its high activity in the kidney may lead to the rapid conversion of ^18^F-DOPA to ^18^F-labeled dopamine, which is then reabsorbed or utilized by the renal tubules. The activity of AADC in the kidney significantly influences the metabolism of L-DOPA. Studies have shown that a high-sodium diet increases AADC enzyme activity in the renal tubules, while a low-sodium diet reduces it (Hayashi et al., 1990). This suggests that the activity of the AADC metabolic enzyme in the kidney may be regulated by renal sympathetic nerve activity. Additionally, RDN may affect renal sympathetic nerve function by altering other key factors in the renal dopaminergic system.

This hypothesis provides a new direction for future research: further investigation into the effects of RDN on the renal sympathetic and dopaminergic systems will help deepen our understanding of the molecular mechanisms underlying RDN’s treatment of hypertension and other related diseases.

## Data Availability

The original contributions presented in the study are included in the article/Supplementary Material, further inquiries can be directed to the corresponding authors.
