# Supplementary material for: 18F-DOPA uptake illustration acts as an indicator for renal sympathetic activity
Source: Front Physiol. 2025 Oct 20;16:1569699. doi: 10.3389/fphys.2025.1569699 (PMC12580137; doi:10.3389/fphys.2025.1569699)
Supplement: Supplementary file 1 [file Table1.docx]

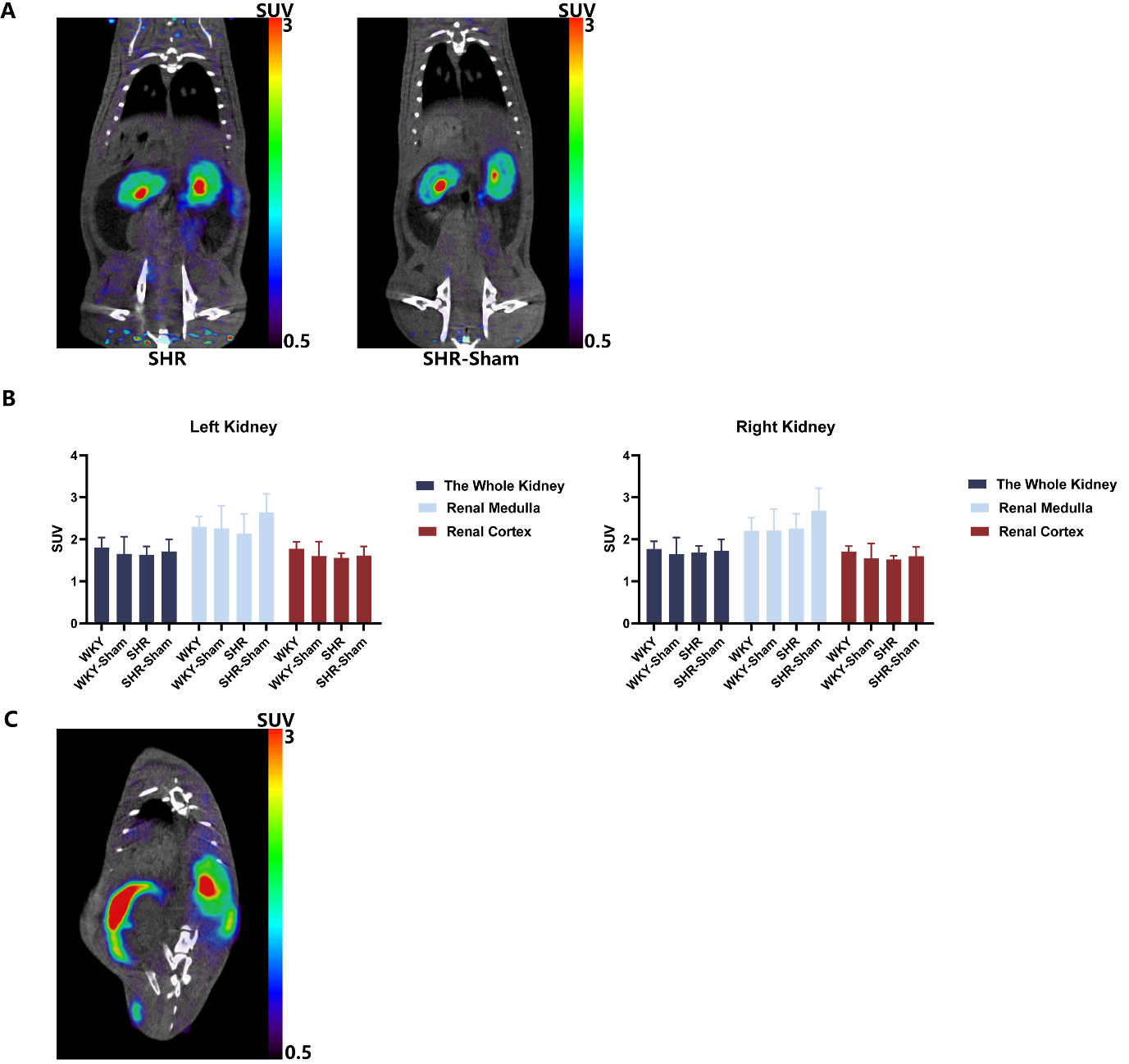


**Supplementary Figure 1. Renal uptake of ^18^F-DOPA in WKY-Sham and SHR-Sham.** (A) Representative ^18^F-DOPA microPET-CT images. The images display renal uptake of ^18^F-DOPA in SHR rats before and after sham surgery. No significant differences in ^18^F-DOPA distribution within the kidneys are observed following the sham procedure. (B) Statistical analysis of the standard uptake values of ^18^F-DOPA in the kidneys of WKY rats and SHR rats before and after sham surgery. After sham surgery, no significant change in renal ^18^F-DOPA uptake was observed in both WKY and SHR groups compared to baseline. one-way ANOVA. Data are presented as mean ± SD. Each group: n=4. (C) Abnormal kidney imaging in some rats. Due to potential surgical factors, several rats displayed markedly abnormal kidney images on CT, with significant swelling, enlargement, and displacement of the affected kidney. These data were excluded from the statistical analysis.
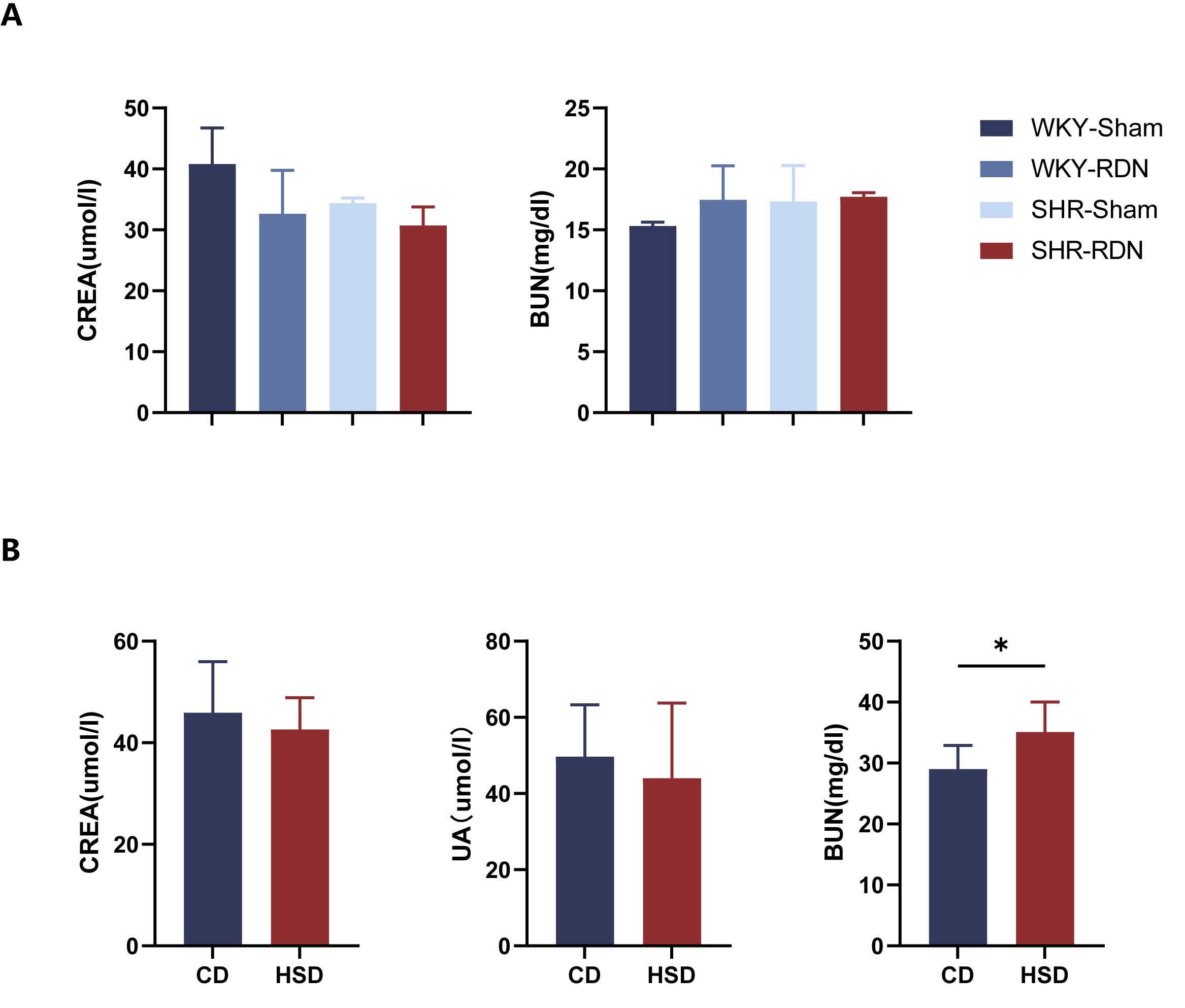


**Supplementary Figure 2. Serum renal function in all rats undergoing ^18^F-DOPA PET-CT scanning.** (A) Comparison of serum renal function indicators. No significant difference in serum renal function indicators was observed among the WKY-Sham, WKY-RDN, SHR-Sham, and SHR-RDN groups of rats. Each group: n=3. one-way ANOVA. Data are presented as mean ± SD. (B) UREA, CREA, and BUN levels in CD and HSD rats. No significant difference in serum UREA and CREA levels was observed between CD and HSD rats, while serum BUN levels were slightly elevated in HSD rats. Each group: n=8.*P<0.05, Student's t-test. Data are presented as mean ± SD.


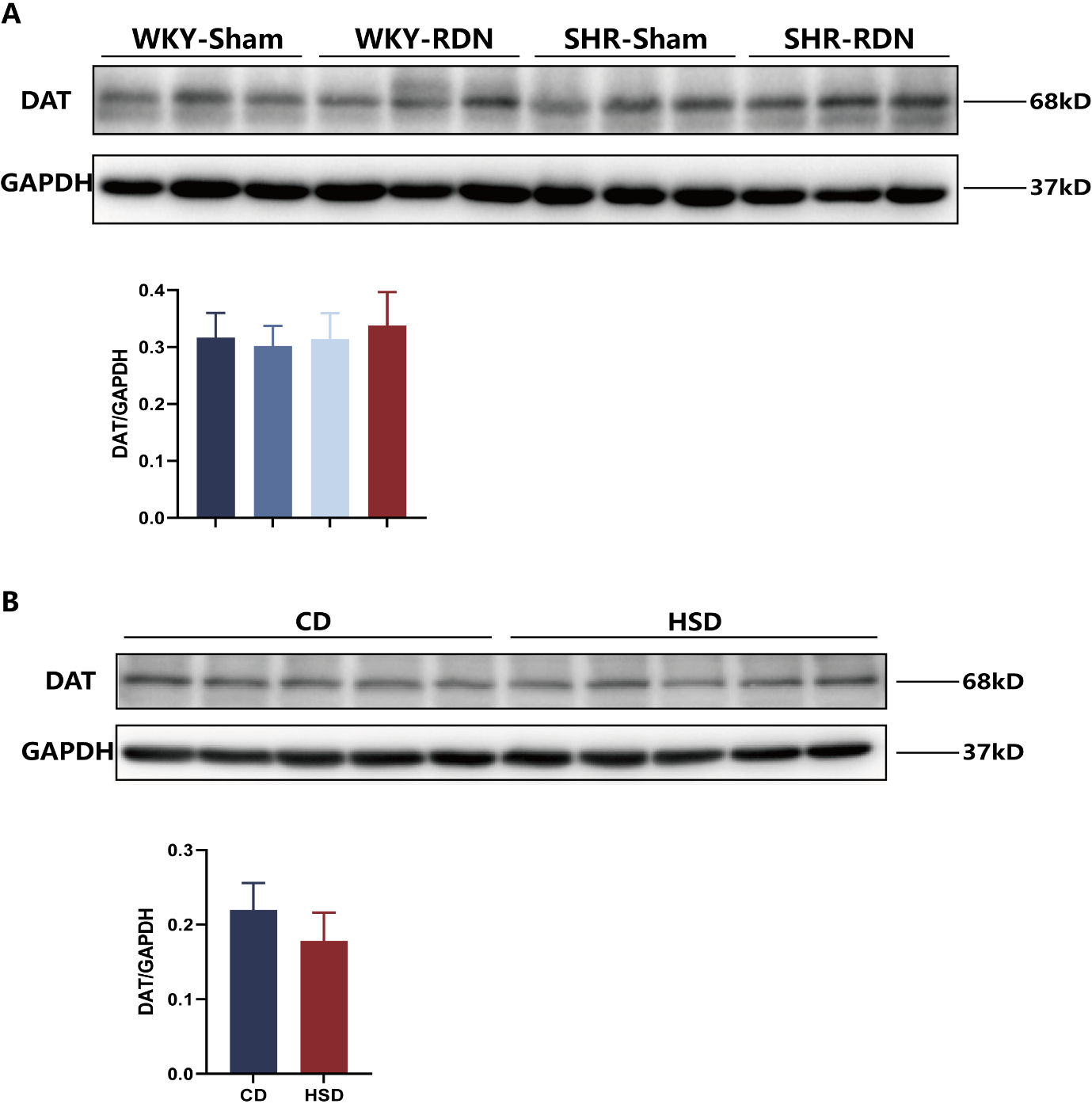


**Supplementary Figure 3. DAT protein expression levels in renal tissue of RDN rats and high-salt diet rats.** (A) DAT protein expression in renal tissue of WKY-Sham, WKY-RDN, SHR-Sham, and SHR-RDN rats. No statistically significant differences in DAT protein expression were observed between these groups, indicating a similar pattern of expression across the experimental conditions. Each group: n=3. ANOVA. Data are presented as mean ± SD. (B) DAT protein expression in renal tissue of CD and HSD rats. Similarly, no statistically significant differences in DAT protein expression were detected between control diet (CD) and high-salt diet (HSD) rats, suggesting that high-salt feeding did not notably affect the expression of DAT in the kidneys. Each group: n=5. Student's t-test. Data are presented as mean ± SD.
